# Supplementary material for: Bayesian refinement of protein structures and ensembles against SAXS data using molecular dynamics
Source: PLoS Comput Biol. 2017 Oct 18;13(10):e1005800. doi: 10.1371/journal.pcbi.1005800 (PMC5662244; doi:10.1371/journal.pcbi.1005800)
Supplement: S1 Archive — (BZ2) [file pcbi.1005800.s011.tar.bz2 › gromacs_4.62_bayesian_saxs_refi/share/html/online/map.html]

map file format


## map file format

|  |  |
| --- | --- |
| Main Table of Contents | **VERSION 4.6** |
| GROMACS homepage | **Sat 19 Jan 2013** |

---

### Description

This file maps matrix data to RGB values which is used by the
do\_dssp program.

The format of this file is as follow: first line number of elements
in the colormap. Then for each line: The first character is
a code for the secondary structure type.
Then comes a string for use in the legend of the plot and then the
R (red) G (green) and B (blue) values.

In this case the colors are
(in order of appearance): white, red, black, cyan, yellow, blue,
magenta, orange.

```
8
~  	Coil		1.0	  1.0	  1.0
E 	B-Sheet		1.0	  0.0	  0.0
B 	B-Bridge	0.0	  0.0	  0.0
S 	Bend		0.0	  0.8	  0.8
T 	Turn		1.0	  1.0	  0.0
H 	A-Helix		0.0	  0.0	  1.0
G 	3-Helix		1.0	  0.0	  1.0
I 	5-Helix		1.0	  0.6	  0.0
```

---

http://www.gromacs.org
